# A review of the bioeffects of low-intensity focused ultrasound and the benefits of a cellular approach

**DOI:** 10.3389/fphys.2022.1047324

**Published:** 2022-11-10

**Authors:** Morgan N. Collins, Karen A. Mesce

**Affiliations:** ^1^ Graduate Program in Neuroscience, University of Minnesota, Saint Paul, MN, United States; ^2^ Department of Entomology and Graduate Program in Neuroscience, University of Minnesota, Saint Paul, MN, United States

**Keywords:** low-intensity focused ultrasound, non-invasive brain stimulation, neuromodulation, transcranial focused ultrasound, leech, thermal modulation, model organisms, mechanotransduction

## Abstract

This review article highlights the historical developments and current state of knowledge of an important neuromodulation technology: low-intensity focused ultrasound. Because compelling studies have shown that focused ultrasound can modulate neuronal activity non-invasively, especially in deep brain structures with high spatial specificity, there has been a renewed interest in attempting to understand the specific bioeffects of focused ultrasound at the cellular level. Such information is needed to facilitate the safe and effective use of focused ultrasound to treat a number of brain and nervous system disorders in humans. Unfortunately, to date, there appears to be no singular biological mechanism to account for the actions of focused ultrasound, and it is becoming increasingly clear that different types of nerve cells will respond to focused ultrasound differentially based on the complement of their ion channels, other membrane biophysical properties, and arrangement of synaptic connections. Furthermore, neurons are apparently not equally susceptible to the mechanical, thermal and cavitation-related consequences of focused ultrasound application—to complicate matters further, many studies often use distinctly different focused ultrasound stimulus parameters to achieve a reliable response in neural activity. In this review, we consider the benefits of studying more experimentally tractable invertebrate preparations, with an emphasis on the medicinal leech, where neurons can be studied as unique individual cells and be synaptically isolated from the indirect effects of focused ultrasound stimulation on mechanosensitive afferents. In the leech, we have concluded that heat is the primary effector of focused ultrasound neuromodulation, especially on motoneurons in which we observed a focused ultrasound-mediated blockade of action potentials. We discuss that the mechanical bioeffects of focused ultrasound, which are frequently described in the literature, are less reliably achieved as compared to thermal ones, and that observations ascribed to mechanical responses may be confounded by activation of synaptically-coupled sensory structures or artifacts associated with electrode resonance. Ultimately, both the mechanical and thermal components of focused ultrasound have significant potential to contribute to the sculpting of specific neural outcomes. Because focused ultrasound can generate significant modulation at a temperature <5°C, which is believed to be safe for moderate durations, we support the idea that focused ultrasound should be considered as a thermal neuromodulation technology for clinical use, especially targeting neural pathways in the peripheral nervous system.

## Introduction

Focused ultrasound (FUS) neuromodulation uses high frequency sound (>20 kHz, the upper limit of sound frequencies audible to humans) to modulate neuronal activity. It is an emerging technology with tremendous clinical potential for the treatment of neurological disorders. Interest in FUS neuromodulation has soared in recent years, buoyed by the success of implantable neuromodulation technologies including deep brain stimulation, which has proven therapeutic in treating disorders ranging from epilepsy to Parkinson’s disease (Miocinovic et al., 2013). Unlike other noninvasive technologies including transcranial magnetic stimulation (Deng et al., 2013) and transcranial direct current stimulation (Neuling et al., 2012), FUS is able to deliver energy noninvasively to deep brain areas with spatial specificity on the order of millimeters (Hynynen and Clement, 2007; Leo et al., 2016, 2018), sparing future patients the risks and financial burdens associated with surgical placement of implanted devices. Though the use of FUS in combination with non-endogenous ion channels is currently under investigation [“sonogenetics” (Ibsen et al., 2015; Ye et al., 2018)], FUS neuromodulation does not require the heterologous expression of proteins a la optogenetics (Fenno et al., 2011), and thus avoids subjecting patients to genetic manipulation. In sum, FUS’s precise yet noninvasive nature yields strong advantages over current neuromodulatory technologies, and the technique merits intensive investigation on a basic level (the subject of this review), as well as synergistic development on a clinical level.

## Historical perspectives

Ultrasound is a natural phenomenon; sounds at frequencies up to ca. 200 kHz are utilized by a diverse array of animal species to communicate with their young (Portfors and Perkel, 2014), detect prey (Jones and Holderied, 2007), evade predators (Kawahara and Barber, 2015), and navigate (Au, 1993). The origins of man-made ultrasound date to the Curie brothers’ 19th century discovery of piezoelectricity, a concept wherein the application of pressure to some materials including quartz generates an electrical potential (and its reverse: application of a potential generates pressure) (Newman and Rozycki, 1998). The development of ultrasound technology was subsequently accelerated during the first world war, fueled by demand for SONAR-based submarine detection (Manbachi and Cobbold, 2011).

The effects of ultrasound on living organisms were first documented in 1927, wherein its application for several minutes was found to be lethal to “lower forms of life” including fish and frogs (Wood R. W. and Loomis A. L., 1927; Wood RW. and Loomis AL., 1927). The following year, the first examination of ultrasound’s effects on nervous tissue was undertaken, and it was reported that ultrasound was unable to stimulate frog sciatic nerves (Harvey and Loomis, 1928). Intensive study of FUS’s effects on the nervous system began in the 1950s by W.J. Fry, F.J. Fry, and others. Reports from this era included descriptions of FUS-induced suppression of neural firing in the crayfish ventral nerve cord and frog spinal cord (Fry et al., 1950; Wulff et al., 1951), and reduction of amplitude of visually evoked potentials following sonication of the lateral geniculate nucleus in cats (Fry et al., 1958).

Early reports of FUS’s excitatory actions date to the 1970s. This work, spearheaded by L.R. Gavrilov and others, demonstrated the technology’s ability to activate mechanosensory structures when targeting human skin (Gavrilov et al., 1977a) and isolated Pacinian corpuscles from cats (Gavrilov et al., 1977b). FUS was also found to evoke auditory potentials in frogs (Gavrilov et al., 1977b), cats (Wiederhold, 1978), and humans (Tsirulnikov et al., 1988).

Non-sensory FUS-induced excitation was described in the 1980s, when a report was published describing stimulation of non-sensory mammalian cortex in cats and rabbits (Velling and Shklyaruk, 1988). In the last 15 plus years, this idea has gained tremendous traction as interest in FUS neuromodulation has surged, though the ability of FUS to activate non-sensory structures remains a matter of debate.

## Focused ultrasound effect direction

The current renaissance of FUS neuromodulation has yielded dozens of publications describing effects on an ever-increasing array of animal species (invertebrate, amphibian, mammal) in diverse paradigms (transcranial, peripheral nerve, cell culture, slice, *etc.*). Despite this enormous collective undertaking, researchers have thus far failed to reach a consensus regarding the technology’s most critical element; that is, whether FUS induces neuronal excitation or inhibition (Dell'Italia et al., 2002). The following section provides an overview of the FUS neuromodulation literature to date. Studies employing high-intensity focused ultrasound (HIFU) have been omitted here. Although some researchers have found success in blocking action potential conduction with HIFU (Foley et al., 2008; Lee et al., 2015a,b), it is more commonly used for destructive applications (e.g., ablating tissue) (Elhelf et al., 2018). Most neuromodulation researchers have opted to use less destructive low-intensity FUS that falls within the FDA-permissible range for non-ophthalmic diagnostic applications (spatial peak pulse average intensity (I_SPPA_) of ≤190 W/cm^2^) (FDA, 2019); this work is discussed below.

### Transcranial focused ultrasound brain studies

Transcranial FUS (tFUS) applications have been the subject of enthusiastic research and a number of reviews, and thus its discussion here will be limited. Clearly, there exists tremendous demand for noninvasive neuromodulatory therapies to normalize pathological aberrant firing in cortical [e.g., epilepsy (Chevassus-Au-Louis et al., 1999)] and subcortical [e.g., Parkinson’s disease (Galvan and Wichmann, 2008)] brain areas. Output metrics vary, but are commonly tFUS-induced changes in the amplitude of sensory-evoked potentials (SEPs) (Kim et al., 2012; Legon et al., 2014; Chu et al., 2015; Kim et al., 2015; Legon et al., 2018a; Legon et al., 2018b; Darrow et al., 2019), or motor responses (Tufail et al., 2010; Kim et al., 2012; King et al., 2013; Younan et al., 2013; Mehić et al., 2014; Kamimura et al., 2015; Lee et al., 2016). The majority of studies examining effects on the SEPs report reductions in amplitude; these include reduction of somatosensory-evoked potentials in rats (Chu et al., 2015; Darrow et al., 2019) and humans (Legon et al., 2014; 2018a), and visually-evoked potentials in rats. By contrast, studies examining motor responses typically report excitation, including the elicitation of limb movements in mice (Younan et al., 2013; Mehić et al., 2014; Kamimura et al., 2016) and sheep (Lee et al., 2016). In the context of the diverse outcomes and associated underlying mechanisms of tFUS, there is a continuing need to separate out the short-term effects of tFUS from the longer-term changes in homeostatic plasticity and gene regulation.

### Peripheral mammalian studies

In addition to the aforementioned transcranial studies, there is enthusiasm regarding FUS’s potential as a peripheral neuromodulatory therapy. Invasive peripheral nerve stimulation is currently utilized in the treatment of chronic pain (Chakravarthy et al., 2016); FUS could potentially provide a noninvasive alternative to implantable devices. Several mammalian peripheral studies have explored effects on the sciatic nerve, though reported outcomes have varied. One group reported that FUS applied to the sciatic nerve evoked muscle activity in mice (Downs et al., 2018), while another, also in mice, found that FUS was unable to evoke potentials, though it did increase the conduction velocity of single units (Ilham et al., 2018). Recently, guinea pig sciatic nerves, *in vivo*, were found to be inhibited *via* an FUS-induced thermal mechanism (Guo et al., 2022).

Cranial nerves have also been targets of attempted FUS modulation. One group transcranially targeted the abducens nerve in rats, and were successful in eliciting abductive eye movements (Kim et al., 2012). Another group working in rats targeted the vagus nerve, and reported a predominately inhibitory effect (Juan et al., 2014). Efforts to modulate the vagus nerve *via* FUS may prove especially fruitful, as implantable vagus nerve stimulators have demonstrated the therapeutic potential for the treatment of several neurological and inflammatory disorders, and are currently FDA-approved for the treatment of epilepsy and depression (Johnson and Wilson, 2018).

### *In vitro* studies

Determining effect direction in intact systems, particularly with respect to transcranial studies, can be confounded by factors including skull reflection and incidental activation of mechanosensitive sensory structures, including auditory hair cells in the cochlea. The former can cause unintended delivery of FUS to off-target areas, particularly in small animals (Younan et al., 2013). The latter has been demonstrated to generate broad cortical activation independent of focus location in guinea pigs (Guo et al., 2018). A similar result was reported in mice (Sato et al., 2018); these mice were also found to exhibit auditory-based startle reflexes in response to FUS application, a finding with implications for studies that report FUS-induced elicitation of movement (Younan et al., 2013; Mehić et al., 2014; Kamimura et al., 2016; Lee et al., 2016).

Researchers seeking to clarify the effect direction of FUS, and its mechanisms of action have attempted to circumvent these confounding constraints through the use of *in vitro* mammalian preparations or invertebrate models. With respect to *in vitro* preparations, groups have described effects in cultured primary neurons and slice preparations, though the directions of these reported effects are inconsistent.

Several studies have examined effects in rodent hippocampal slice preparations. One group reported that FUS elicited Na^+^ and Ca^2+^ transients and evoked action potentials in CA1 neurons in mice (Tyler et al., 2008). Another group reported FUS-induced reduction in evoked fiber volley and dendritic potentials of CA1 neurons from rats (Rinaldi et al., 1991). A group recording from the dentate gyrus of rats, found response direction varied with respect to hippocampal sublayer; fiber volleys and cell bodies were inhibited, but dendritic potentials were enhanced (Bachtold et al., 1998), thus opening up an additional dialogue about FUS direction and its correlation with site-specific membrane excitability.

The few reports of neuromodulation outcomes in cultured neurons have largely described FUS-driven excitation. Cultured primary neurons from embryonic rats were reported to display increased firing when targeted with FUS (Khraiche et al., 2017). Others found that FUS induced increases in neuronal activity in mouse primary neurons as measured by an increase in c-Fos (Qiu et al., 2019). One group found FUS elicited action potentials in cultured hippocampal neurons that heterologously expressed a mechanosensitive bacterial ion channel; however, this effect was absent in the wild-type control cells (Ye et al., 2018).

### Amphibian studies

The earliest nervous tissue exposed to FUS in a laboratory environment was from the frog (Harvey, 1929). In recent years, several groups have examined effects of FUS on the frog sciatic nerve. One group reported FUS-induced inhibition attributed to conduction block (Colucci et al., 2009), while two others reported both neuronal enhancement and suppression, with outcomes biased by parameters (Mihran et al., 1990; Tsui et al., 2005).

In salamanders, two papers have described the effects of FUS on the *ex vivo* retina. FUS was found to indirectly stimulate retinal ganglion cells *via* activation of photoreceptors and post-photoreceptor interneurons (Menz et al., 2013, 2019).

### Invertebrate studies

Explorations of FUS’s effects on invertebrate nervous systems date back to at least the 1960s (Lele, 1963), but the last seven plus years have produced a flurry of FUS publications utilizing “simpler” systems, which offer greater accessibility and fewer regulatory constraints. These systems typically contain far fewer neurons than mammalian models, and their neurons—many of which can be identified across specimens—are often highly stereotyped with respect to their membrane and synaptic properties. These characteristics aid investigators in reducing experimental variability associated with, for example, incidental targeting of different sub-populations of cells across preparations, which could account for some of the differences in reported outcomes among researchers studying mammalian systems. Unfortunately, despite these advantages, the inconsistencies in effect direction present in the mammalian FUS neuromodulation literature are similarly present in the invertebrate literature.

A number of compelling studies have examined the effects of FUS on the nematode *C. elegans*. One group reported that wild-type *C. elegans* were insensitive to low-intensity FUS, though heterologous expression of a mechanosensitive ion channel sensitized neurons to FUS, causing the mutant strain to exhibit behavioral responses following its application (Ibsen et al., 2015). A second group failed to replicate this finding, but reported that wild-type nematodes did respond to FUS perturbation, and this behavioral response was dependent on the expression of a mechanosensitive ion channel involved in touch sensation (Kubanek et al., 2018). A third group similarly found that FUS was able to initiate a behavioral response by activating a mechanosensitive cell (Zhou et al., 2017).

Another more accessible model preparation is the giant axon system of the earthworm. One study found that FUS application to the giant fibers of the nerve cord was effective in eliciting trains of action potentials; however, this effect was mentioned to be indirect and likely *via* FUS stimulation of afferent input (Vion-Bailly et al., 2019, 2022). Another group reported an FUS-induced reduction in action potential amplitude and conduction velocity following sonication, and although a second group, who targeted the medial and lateral giant fibers, could replicate this reduction in action potential amplitude, they did not observe a decrease in conduction velocity (Yoo et al., 2017).

**FIGURE 1:**
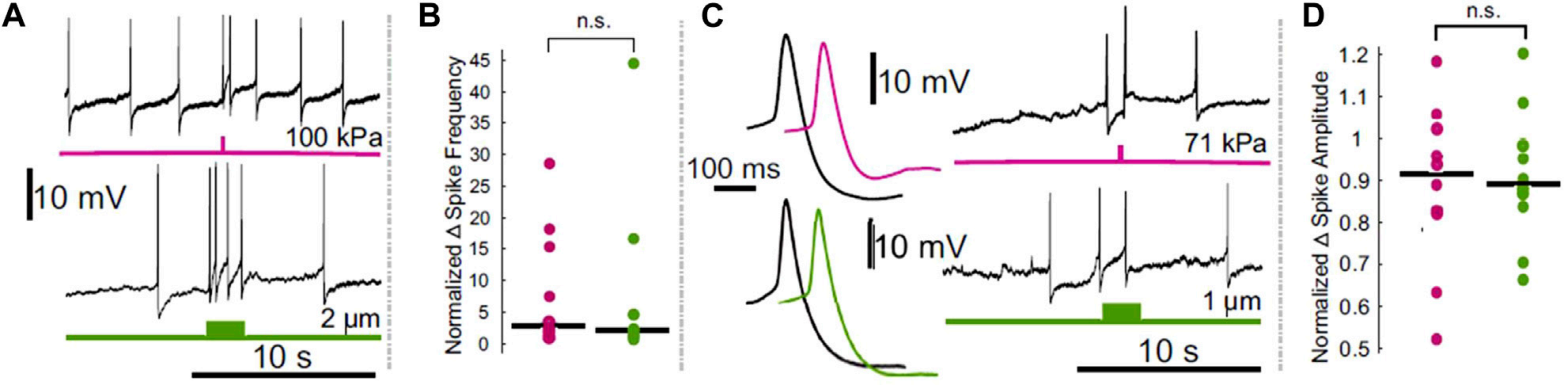
Comparison of the effects of electrode displacement on the spike frequency and amplitude of leech Retzius neurons. **(A)** Intracellular recordings demonstrating ultrasound (upper, pink) and electrode-displacement (lower, green) associated increase in spike frequency. **(B)** Scatter plots comparing the normalized change in spike frequency, during the period of peak effect, in ultrasound (pink) and electrode displacement (green) conditions. Horizontal lines denote medians. The difference between the two did not reach the threshold for significance (Z = 0.1890, *p* = 0.8501, Wilcoxon rank-sum test). **(C)** Intracellular recordings showing that ultrasound (pink) and electrode displacement (green) induce reductions in spike amplitude. Averaged spike waveforms (left) demonstrate reduction in spike amplitude (black waveforms = averaged from the 2 spikes prior to stimulus onset, pink and green waveforms = averaged from the 2 spikes fired during the peak effect period following ultrasound application and electrode displacement, respectively). **(D)** Scatter plots comparing normalized change in spike amplitude during peak effect period in ultrasound (pink) and electrode displacement (green) conditions. Horizontal lines denote medians. The difference between the two did not reach the threshold for significance [*t* (17.3329) = 0.2777, *p* = 0.7845, Welch’s *t*-test]. Figure modified from Collins and Mesce, 2020.

Others have published a series of papers documenting the effects of FUS on the crab leg nerve. Each describes the ability of FUS to stimulate *ex vivo* nerves, including by the generation of *de novo* action potentials (Wright et al., 2015, 2017; Wright, 2016). Excitation was also reported in a crayfish paradigm, in which FUS was found to depolarize an isolated motor axon (Lin et al., 2019). In the leech, it was found that FUS could elicit action potentials in a sensory neuron (Dedola et al., 2020); however, a subsequent study by us indicated that these FUS-mediated action potentials were most likely a result of artifactual electrode resonance (Collins and Mesce, 2020; Figure 1).

### The role of parameters in biasing effect direction

The parameter space for FUS is immense, and an exhaustive discussion of the myriad parameter combinations that have been employed in neuromodulation studies is beyond the scope of this review (for reference, these combinations were recently reviewed (Pasquinelli et al., 2019; Dell’Italia et al., 2022). The following paragraphs provide a brief introduction to ultrasound parameters and their relation to the bioeffects of FUS on tissue, as well as how they factor into safety regulations governing clinical applications. Among the few studies that have reported both excitatory and inhibitory outcomes, several authors have cited parameter selection as a contributing factor to the resultant effect direction (Velling and Shklyaruk, 1988; Tsui et al., 2005; Juan et al., 2014; Kim et al., 2015).

### Mechanical index

FUS waveforms are characterized by two key variables: frequency (cycles per second, measured in Hz) and pressure amplitude (measured in Pa). Pressure oscillates from positive (compresses tissue) to negative (expands tissue) with each cycle of FUS. Negative pressure applied to a fluidic medium (e.g., most body tissues) can generate cavitational bubbles from dissolved gasses within this medium. These microbubbles, typically several microns in diameter, oscillate in size and can collapse (inertial cavitation), resulting in destructive mechanical stress and localized heating (Azhari, 2010). Cavitation increases with increasing peak negative pressure and decreases with frequency. This relationship is described by the Mechanical Index (MI), a measure of cavitation risk. For non-ophthalmic diagnostic applications, FDA guidelines require MI to be less than or equal to 1.9 (FDA, 2019).

### I_SPPA_

FUS is frequently pulsed in neuromodulatory contexts to reduce the risk of rapid heating, which can occur with continuous applications of ultrasound. The number of pulses per second is the pulse repetition frequency (PRF, measured in Hz), and the pulse duration (PD) is the duration in seconds of each pulse. The duty cycle, or percentage of time ultrasound is actively delivered during the application period, is the PRF x PD.

Ultrasound intensity is a measure of the amount of energy delivered to tissue. One common metric reported by FUS neuromodulation researchers is the spatial peak pulse average intensity (*I*_*SPPA*_), a measure of the average intensity of a single ultrasound pulse at the location of peak pressure within the ultrasound focus. The *I*_*SPPA*_ varies by tissue type, dependent on the speed of sound in the targeted tissue and the tissue density. For reference, the speed of sound in human nervous tissue is approximately 1,500 m/s; the density of nervous tissue is approximately 1.06 g/cm^3^ (Azhari, 2010). FDA-permissible *I*_*SPPA*_ for non-ophthalmic diagnostic applications is ≤190 mW/cm^2^.

Another commonly reported intensity metric is the spatial peak time averaged intensity (*I*_*SPTA*_), the average intensity of the pulse repetition period. The *I*_*SPTA*_ is equal to *I*_*SPPA*_ x duty cycle. Higher *I*_*SPTA*_ yields greater tissue heating. FDA-approved upper limits for *I*_*SPTA*_ vary by tissue type, from 720 mW/cm^2^ for peripheral vessel applications, to 94 mW/cm^2^ for cephalic (adult and fetal) applications (FDA, 2019).

Parameter selection can bias the type of bioeffects induced by FUS in targeted tissue. Higher frequencies are associated with greater tissue heating (though this effect is nonlinear, and dependent on tissue type), as are higher intensities and longer periods of acoustic radiation (Azhari, 2010). Cavitation increases with pressure and decreases with frequency (see MI equation). Importantly, heat and mechanical stress can each induce a wide-range of direct and indirect effects on nervous tissue, many of which could result in an increase or decrease in neuronal firing.

## Mechanical gating of ion channels

Many ion channels have mechanosensitive properties. While this property is well-established for classes of channels involved in the transduction of sensory stimuli [e.g., TRP sub-types (Christensen and Corey, 2007) and Piezo (Coste et al., 2010)] and likely ASIC3 channels (Lee and Chen, 2018), evidence is accumulating that other types of ion channels, including voltage-gated ion channels, also have some degree of mechanosensitivity. FUS activation of voltage-gated sodium channels, of particular interest to neuromodulation researchers given their role in generating the rising phase of the action potential, has been cited as a potential driver of FUS-induced neuronal excitation (Tyler et al., 2008; Tufail et al., 2010; Wright et al., 2015; Kubanek et al., 2018). These channels have known mechanosensitive properties (Morris and Juranka, 2007), and prior work has shown that FUS increases channel conductance when heterologously expressed in *Xenopus* oocytes (Kubanek et al., 2016). At present, however, evidence remains weak that FUS activation of voltage-gated sodium channels is an effective actuator of excitation in neurons. In crayfish axons, FUS-induced depolarization persisted following application of the channel blocker TTX (Lin et al., 2019). Additionally, in cultured mammalian neurons, high frequency FUS (43 MHz) was not sufficient to activate sodium channels in a patch clamp preparation (Prieto et al., 2018). Assessing the precise contribution of these channels to FUS-induced increases in firing rate remains difficult, as channel blockers concurrently block neuronal firing, and different types of sodium channels will vary depending on the animals species and tissues tested.

Another family of channels that has been implicated in FUS neuromodulation is the two-pore potassium channel (K2P), a family of potassium-permeable leak channels. Subtypes TRAAK, TREK-1 and TREK-2 are highly mechanosensitive, widely expressed in the CNS, and display increased conductance in response to changes in membrane tension induced by sub atmospheric pressure and laminar stress (Enyedi and Czirják, 2010; Sorum et al., 2021). FUS has been shown to increase conductance of these channel subtypes when expressed in *Xenopus* oocytes (Kubanek et al., 2016) and in cortical slices (Sorum et al., 2021). Increases in K2P conductance hyperpolarizes neurons; effects on these channels could thus contribute to FUS-induced inhibition.

Perhaps, unsurprisingly, FUS has also been shown to activate canonical mechanosensitive ion channels. In cultured mammalian neurons, FUS increased conductance of Piezo1, a channel believed to be a primary actuator of somatosensory mechanotransduction (Prieto et al., 2018). The broader contribution of Piezo channels to FUS-induced neuronal excitation, however, remains unclear. Although these channels are highly expressed in sensory neurons, including those in dorsal root ganglia, expression in the CNS is at least 10-fold lower (Coste et al., 2010).

In *C. elegans*, a behavioral response to FUS has been shown to be dependent on expression of MEC-4, a pore-forming component of a mechanosensitive channel expressed by sensory neurons and belonging to the DEG/ENaC/ASIC family (Kubanek et al., 2018). Most mammalian members of this voltage-independent, sodium-selective channel family are expressed primarily in sensory neurons and are believed to contribute to somatosensation (Eastwood and Goodman, 2012). Acid-sensing ion channels (ASICs), however, are broadly expressed in the CNS (Boscardin et al., 2016). DEG/ENaC/ASIC channels have largely conserved sequences and highly similar structures (Eastwood and Goodman, 2012); it is entirely probable that FUS is able to activate mammalian channels in a manner comparable to its activation of an invertebrate homolog. Although activation of members of this channel family, particularly ASICs, may contribute to FUS-induced neuronal excitation, whether it is desirable to target these channels (e.g., *via* specialized parameters) is another matter as ASIC hyperactivity is implicated in the pathology of inflammatory neurological disorders including pain and neurodegenerative disease (Boscardin et al., 2016). Regardless, FUS-related studies targeted to understand their specific activation are clearly worthy of study.

With respect to FUS activation of other sensory ion channels, a brief mention of the transmembrane channel-like family (TMC) is warranted. To the best of our knowledge, no studies have directly examined the effects of FUS on these channel-like proteins *via* two-electrode voltage clamp in *Xenopus* oocyte expression systems or elsewhere. Two isoforms of this family, TMC1 and TMC2, are believed to transduce sound stimuli following deflection of the tip links on auditory hair cells in the cochlea (Delmas and Coste, 2013). This is highly relevant to *in vivo* mammalian FUS studies, as FUS has been shown to activate auditory hair cells, in turn causing widespread cortical activation (Guo et al., 2018; Sato et al., 2018). Activation of TMC proteins, the likely actuators of sound transduction, may thus contribute to cortical excitation reported in *vivo* studies.

### Intra- and extracellular cavitation

Another popular theory of FUS’s excitatory mode of action is cavitation. Intramembrane cavitation, the effects of which have been described by a “bilayer sonophore” model, is proposed to induce excitation *via* FUS-induced cyclic expansions and contractions of sonophores in the intramembrane space, which in turn modulate membrane capacitance (Krasovitski et al., 2011). Changes in membrane capacitance are predicted to alter ionic currents, resulting in depolarization of the resting membrane potential and a corresponding increase in firing (Plaksin et al., 2014). To the best of our knowledge, FUS-induced intramembrane cavitation-driven neuronal excitation has yet to be empirically demonstrated in neurons.

Cavitation has also been proposed to enact modulation extracellularly by inducing membrane stretch (*via* microstreaming drag, direct jetting or radiation force), which is thought to increase the conductance of ion channels (Wright et al., 2017). Several invertebrate studies have reported results consistent with an extracellular cavitation mechanism. In crab axons, for example, neurostimulation was found to require high pressures, occur as an “all or nothing” phenomenon (consistent with sporadic formation of microbubbles), and occasionally induce tissue damage, as is known to accompany inertial cavitation (Wright et al., 2017). The same group also reported that stimulation in this system occurs in concert with stable or inertial cavitation as measured with a Passive Cavitation Detector, and does not occur in its absence (Wright, 2016). In earthworms, researchers hypothesized a cavitation-based mechanism, and found that stimulation of the medial and lateral giant fibers was most successful at cavitation-promoting parameters (e.g. higher pressures) (Vion-Bailly et al., 2019). It should be noted, however, that they utilized pressures in excess of those used in the majority of mammalian studies reporting FUS-induced excitation.

## Thermal effects

**FIGURE 2:**
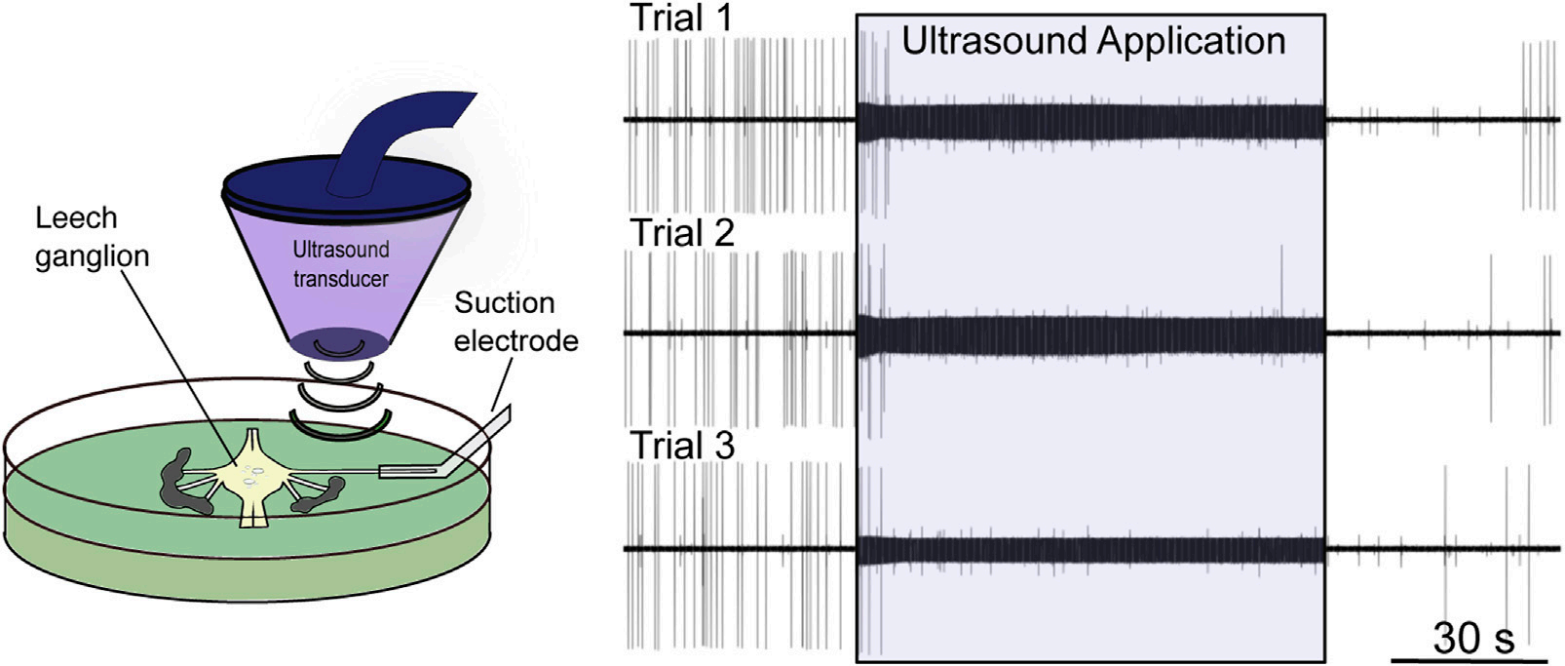
Low-intensity focused ultrasound neuromodulation of motoneuron DE-3. *Left*: Stylized schematic of experimental design showing an isolated single leech ganglion and its dorsal posterior (DP) nerve pinned out in a Petri dish. The ultrasound transducer is depicted, which generates and focuses the ultrasound on the DP nerve. The firing rate of the DE-3 motoneuron, which sends its axon through the DP nerve, is recorded *via* a suction electrode. *Right*: Ultrasound (960 kHz) induces repeatable, reversible inhibition of DE-3 spiking across multiple trials of ultrasound application. Figure modified from Mesce and Newhoff, 2020, with permission from Elsevier copyright 2020.

**FIGURE 3:**
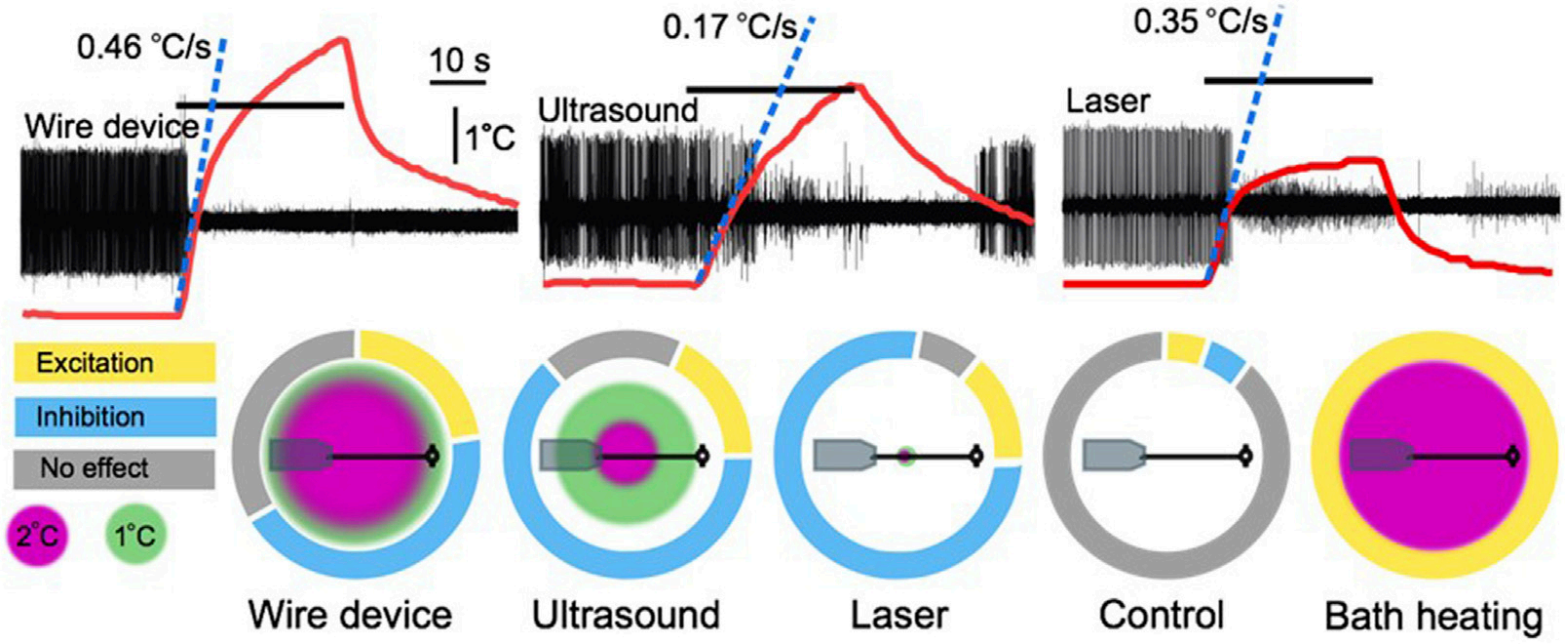
A comparison of the thermal effects of a wire device, ultrasound, a 50 mW laser, and bath heating. Upper traces are extracellular leech nerve recordings that demonstrate inhibition of an identified motoneuron (largest unit) induced by 30 s applications of the three stimuli. Overlaid on the traces are thermocouple recordings demonstrating the height and rate of heating of each apparatus. Lower charts demonstrate the spatial profile of the different heating apparatuses overlaid on a scale depiction of a leech ganglion and nerve connected to a suction electrode. Surrounding these depictions are modified pie charts demonstrating the relative proportion of trials in our leech nerve study (Collins et al., 2021) that resulted in excitation, inhibition, or no effect. Figure modified from Collins et al., 2021.

Tissue absorbs ultrasound as heat, itself a potent neuromodulator (Janssen, 1992). The contribution of tissue heating to the neuromodulatory effects of FUS has been a matter of contention since the field’s infancy, with several early groups assuming a thermal mode of action (Lele, 1963; Ueda et al., 1977), and others proposing a nonthermal mechanism (Fry et al., 1950, 1958; Barnard et al., 1955; Takagi et al., 1960). Some early justifications for a nonthermal mechanism stem from assumptions since proven false. One group dismissed the possibility of thermal suppression of firing of the crayfish ventral nerve cord at temperatures in the 1–2°C range; this “slight” temperature increase was believed to be capable only of increasing neural activity (Wulff et al., 1951). It has since been shown that temperature increases close to this range provided *via* FUS application (Darrow et al., 2019; Collins et al., 2021) or other heating modalities (e.g., infrared (Cayce et al., 2011; Lothet et al., 2017)) can, indeed, inhibit neuronal activity (Figure 2). Other groups have argued against a thermal effect by demonstrating that bath heating to equivalent temperatures to those induced by FUS failed to elicit a comparable response (Fry et al., 1950; Fry, 1953; Takagi et al., 1960). More recent work has shown that thermal effects are, in part, dependent on the spatial dimensions of tissue heating (Wells et al., 2007), a factor lost by manipulating broad bath temperature. The study by Collins et al. (2021) further demonstrates the relevance of the spatial distribution of a thermal stimulus (Figure 3).

Heat has been reported to have a wide range of effects on the nervous system, many of which could contribute to excitatory or inhibitory FUS neuromodulation (or both). On a basic level, heat potentiates enzymatic reactions. Many neuronal functions are governed by these reactions, and are thus susceptible to thermal modulation. One example is the N^+^/K^+^-ATPase enzyme that maintains sodium and potassium ion concentration gradients, whose activity is known to increase in hyperthermic conditions (Gorman and Marmor, 1970). This pump exchanges three intracellular sodium ions for two extracellular potassium ions with each cycle (Kaplan, 2002); increasing pump activity thus has a net hyperpolarizing effect, which could inhibit neuronal firing.

Heat is also known to increase the gating kinetics of ion channels (Janssen, 1992), the gross effects of which would depend on the channels most affected. As previously mentioned, voltage-gated sodium channels are commonly cited as potential actuators of FUS neuromodulation (Tyler et al., 2008; Tufail et al., 2010; Wright et al., 2015; Kubanek et al., 2018). The gating of these channels has long been known to be influenced by temperature—as temperature rises, sodium conductance increases, though inactivation kinetics are also increased (Collins and Rojas, 1982). The implications of FUS-induced heat on sodium channels would depend on which effect was dominant—increased sodium conductance would excite neurons, while accelerating channel inactivation would result in inhibition *via* conduction block.

Another channel family implicated in an FUS response, K2P, has members known to be highly thermosensitive. The three channel subtypes shown to be responsive to FUS stimulation (TREK-1, TREK-2, and TRAAK) (Kubanek et al., 2016) are particularly sensitive to changes in temperature. Specifically, heat is known to potentiate the activity of these potassium-permeable leak channels, which inhibits neural activity by hyperpolarizing the resting membrane potential (Schneider et al., 2014).

Finally, heat has also been shown to influence synaptic activity. Heat is believed to act presynaptically by facilitating synaptic vesicle exocytosis, and to exert further influence on neuronal signaling by modulating the diffusion of neurotransmitters in the synaptic cleft (Wang et al., 2014). This is noteworthy, as it remains an open question whether many observed effects of FUS are direct, or result from input from synaptically coupled sensory cells (Guo et al., 2018; Sato et al., 2018). Though heat may not be the primary actuator in the latter instances, it could nonetheless potentiate these synaptic effects.

## The single-cell approach

Despite tremendous efforts by researchers, it remains unclear which mechanism(s) are primarily responsible for observed FUS outcomes, and whether these effects, in the absence of confounding factors (including the activation of sensory structures) are excitatory or inhibitory in nature. Determining this technology’s root effect is prohibitively difficult in intact mammalian systems, in which results may be biased by unintentionally targeting different subpopulations of neurons across preparations, thus incidentally activating mechanosensitive sensory receptors, or causing other off-target effects due to factors including skull reflection.

In recognition of the limitations in their respective intact neural systems, FUS neuromodulation researchers investigating mechanisms of action have applied their efforts to pared down systems, typically cultured mammalian neurons (Rinaldi et al., 1991; Mihran et al., 1996; Prieto et al., 2018; Qiu et al., 2019), mammalian slice preparations (Rinaldi et al., 1991; Bachtold et al., 1998; Tyler et al., 2008; Muratore et al., 2009), or tractable invertebrate models (Wahab et al., 2012; Wright et al., 2015; Zhou et al., 2017; Kubanek et al., 2018; Lin et al., 2019; Vion-Bailly et al., 2019, 2022; Dedola et al., 2020). Isolation in culture is known to alter the intrinsic electrical properties of neurons (Turrigiano et al., 1994); FUS effects on cultured cells may thus differ from outcomes in intact systems. Slice preparations, which maintain some neural circuitry, benefit from “natural” neuronal activity, yet lack experimental flexibility, particularly when compared to highly tractable invertebrate models. Thus, we have adopted this latter strategy by exploring the effects of FUS on identified neurons in the medicinal leech, *Hirudo verbana*. This approach requires sacrificing the use of perfect proxies of intact mammalian neurons (invertebrate neurons, for example, lack myelin), but benefits from a tremendous gain in experimental accessibility. Moreover, the basic properties of most invertebrate neurons closely resemble those in mammalian neurons; invertebrates have been the subject of many seminal investigations that have defined major principles of neuroscience, most notably the determination of the ionic currents underlying the action potential in the squid giant axon (Hodgkin and Huxley, 1952), and the discovery of the molecular basis of learning and memory in the sea slug (Walters et al., 1979; Glanzman, 1995).

### Advantages of the leech in comparison with other systems

The leech is an advantageous animal preparation in which to explore the cellular effects of FUS. As the preceding paragraphs attest, this animal is exceedingly well characterized with respect to identified neurons and their ionic conductances. Additional key advantages are its size, and the ease with which single units from identified neurons can be recorded intra- and extracellularly. While other commonly utilized invertebrate models share some of these attributes, they also present one or more limitations that restrict their utility for usage.

Several prior studies have examined the effects of FUS in *C. elegans* (Ibsen et al., 2015; Zhou et al., 2017; Kubanek et al., 2018), a popular invertebrate model system due to its limited cell number (ca. 302 neurons), relatively simple neural circuitry, and the ease by which it can be manipulated genetically. Despite the completeness of its genome and connectome, its atypical form of neural signaling somewhat limits its relevance. Unlike all vertebrates and most invertebrates (including leech), *C. elegans* neurons do not express voltage-gated sodium channels, nor do they fire sodium-mediated action potentials; neurotransmission occurs *via* graded calcium waves (Bargmann and Kaplan, 1998). Thus, the lack of voltage-gated sodium channels may somewhat lessen the relevance of FUS-related effects reported in this system, as these channels have been hypothesized to be actuators of FUS’s neuromodulatory effects (Tyler et al., 2008; Tufail et al., 2010; Wright et al., 2015; Kubanek et al., 2018). Another limitation in utilizing *C. elegans* in FUS paradigms is its small size (∼1 mm in length). Targeting subpopulations of neurons, much less single cells, is more difficult in this system than in the much larger leech. Consequently, two of the three FUS investigations utilizing this model of which we are aware measured gross behavioral responses to FUS stimuli *versus* individual neuronal responses (Ibsen et al., 2015; Kubanek et al., 2018). Similar size concerns would apply to another popular invertebrate model system, the fruit fly *Drosophila melanogaster*. Recently, whole ganglia from the sea slug *Aplysia californica* were demonstrated to respond to FUS modulation (Jordan et al., 2022). Having large and identifiable neurons, like the leech, this preparation holds future promise in the quest to understand cell-specific membrane-related mechanisms underlying FUS modulation.

The earthworm, *Lumbricus terrestris*, has also been explored as a model system in FUS experiments. The medial and lateral giant axonal fibers of the worm are large and easily accessible, and the animal’s size prevents experimental limitations associated with smaller invertebrates. In contrast to our experiments in the medicinal leech, however, paradigms examining FUS actions have typically required electrically evoking spikes in these fibers (Wahab et al., 2012; Yoo et al., 2017; Vion-Bailly et al., 2019). Despite widespread use of artificial neuronal electrical stimulation in neuroscience studies, evoked activity is believed to differ intrinsically from natural activity (Albensi et al., 2007); FUS effects on the genesis of evoked potentials may not necessarily fully reflect effects in more naturalistic intact systems.

Other groups have reported the effects of FUS on neurons extracted from crabs (Wright et al., 2015, 2017) (*Cancer pagurus*). These experiments have investigated the ability of FUS to evoke and modulate compound action potentials (Wright et al., 2015, 2017). These compound action potentials measure multiunit activity, thus interpreting results of the technology’s effects on a population level can be challenging, as it is difficult to determine whether effects stem from a direct inhibition or excitation of all targeted neurons, or from the selective modulation of a subpopulation of cells, which synaptically influence the activity of the remaining population. Ultimately, this approach lacks the precision of measurement of single units, which can easily be achieved in the leech.

### Contribution of the leech model to understanding the thermal or mechanical modulation of neural activity

A major goal of FUS neuromodulation studies across simpler model systems is to generate knowledge that can eventually be leveraged for the development of human therapies. Consequentially, most intact studies have explored FUS bioeffects on mammalian models. The leech, an annelid, possesses many benefits for use in basic studies of FUS’s actions, including the ability to examine effects on single identified neurons, but its nervous system, like that of other invertebrates, is an imperfect proxy for mammalian systems. Thus, it could be argued that our results in the leech (Collins and Mesce, 2020; Collins et al., 2021) diverge from those observed in mammals because of intrinsic differences between vertebrate and invertebrate nervous systems, although support for this stance is weak.

First, it must be noted that invertebrate and vertebrate neurons share many more similarities than differences. As mentioned previously, much of our current understanding of vertebrate neurophysiology was first described in invertebrates, including the ion currents that govern the generation of the action potential (Hodgkin and Huxley, 1952). The action potential of the leech, too, is governed by a rising phase meditated by voltage-gated sodium channels (Na_V_), and a falling phase mediated by voltage-gated potassium channels (K_V_) (Kleinhaus, 1976; Kleinhaus and Prichard, 1976). This must be noted, as Na_V_ channel types have been implicated in neuronal response to FUS (Tyler et al., 2008; Kubanek et al., 2016; Prieto et al., 2018), yet these channels are not expressed by all invertebrates, including *C. elegans* (Zhou et al., 2017; Kubanek et al., 2018). Other classes of ion channels hypothesized to be mechanically activated by FUS, including two-pore potassium channels (Kubanek et al., 2016; Zhao et al., 2017), transient receptor potential (TRP) channels (Ibsen et al., 2015), and voltage-gated calcium channels (Tyler et al., 2008; Tufail et al., 2010), are expressed in the leech as reported in a published transcriptome (Northcutt et al., 2018). The authors did not explicitly specify the presence of two other types of ion channels that have been hypothesized to underlie FUS’s mechanical effects in other systems. These are members of the degenerin/epithelial Na^+^ channel (DEC/ENaC) family, which have been reported to underlie behavioral responses to FUS in *C. elegans* (Kubanek et al., 2018), and Piezo channels, which have also been reported to respond to FUS (Prieto et al., 2018; Qiu et al., 2019). Expression of DEC/ENaC in the leech is highly likely, as this channel family is broadly conserved across all major animal lineages (Moroz et al., 2014; Lynagh et al., 2018). Similarly, Piezo channels, though not described in the transcriptome, are almost certainly expressed in the leech, as conservation of related channels extends to organisms as distantly related to mammals, like plants and protozoa (Coste et al., 2010). Thus, in the leech, it could be argued that the lack of FUS-induced mechanical modulation of motoneurons is not due to some evolutionarily lack of ion channels necessary to actuate mechanical effects (Collins et al., 2021). Possibly, multiple mechanotransductive proteins are needed in some neurons to form a type of synergy to effectuate an FUS-induced mechanoresponse, as has been recently shown in *C. elegans* (e.g., Magaram et al., 2022).

Leeches and other invertebrate nervous systems also differ from those in mammals with respect to glial cells. Invertebrates lack myelinating glia (oligodendrocytes and Schwann cells), and thus their axons differ anatomically and physiologically from most myelinated axons in mammalian systems (but not unmyelinated ones), a distinction that could underlie our inability to mechanically elicit spikes when targeting a leech nerve (Collins et al., 2021). Ion channel distribution in myelinated axons is highly concentrated to nodes of Ranvier, whereas unmyelinated axons have more diffuse distribution (Waxman and Murdoch Ritchie, 1985). Nodes of Ranvier, sites of Na_V_ density of over 1,200 channels per square micron, are interspersed along myelinated axons at distances approximately 100x axonal diameter (Poliak and Peles, 2003). Most cortical axons are around 500 nm in diameter (Liewald et al., 2014), yielding node spacing of approximately 50 μm. FUS foci, on the order of hundreds of microns to several millimeters in diameter, thus stimulates dozens of nodes in aggregate, as opposed to a singular site of concentrated channels. The extent to which the stimulation of many sites of concentrated channels as opposed to a comparable area of membrane with more uniformly distributed channels contributes to differential outcomes is difficult to predict. This distinction loses relevance, however, when considering that 1) unmyelinated axons are known to innervate the CNS and the periphery of mammals (Liewald et al., 2014), and 2) ion channels in unmyelinated axons in mammals and invertebrates alike are reported to exhibit patterns of clustering to increase the efficiency of action potential conduction in a strategy similar to clustering at nodes of Ranvier (Freeman et al., 2016).

## Parameter-associated limitations as a contributor to the lack of mechanical modulation

Key factors in FUS neuromodulation paradigms are the specific FUS parameters, which include characteristics that define the FUS waveform and its consequential bioeffects including frequency and amplitude (pressure, measured in Pa), and pulse parameters including pulse duration (PD) and pulse repetition frequency (PRF), which further influence FUS intensity and heat output (Constans et al., 2018). In our studies of single identified leech motoneurons, we came to the conclusion that the bioeffects of FUS-mediated neuronal inhibition (*via* action potential blockade) were thermally mediated. A parallel study to ours conducted in mammalian peripheral nerves came to the same conclusion that suppression of neural activity was mediated by FUS thermal effects (Guo et al., 2022). In the following section we will outline the FUS stimulus parameters we used to arrive at our conclusions. The FUS frequency we used, 960 kHz, is higher than most used in transcranial applications (e.g., 250 kHz in monkeys (Yang et al., 2018), 350 kHz in rats (Kim et al., 2015), and 500 kHz in mice (Mehić et al., 2014)). Higher frequencies, however, generate less cavitation, and 960 kHz may thus be too high to generate proposed excitatory cavitational effects (Krasovitski et al., 2011; Plaksin et al., 2014). Although cavitational effects have been visualized *via* electron microscopy in cells treated with 1 MHz FUS (Krasovitski et al., 2011), a comparable frequency (1.1 MHz) was found insufficient to generate cavitation-induced *de novo* action potentials in a paradigm similar to our own (peripheral nerve in crab) (Wright et al., 2017).

Another parameter that may have influenced our inability to mechanically modulate neural activity is our FUS pressure (Collins et al., 2021). We applied pulses with 660 kPa amplitude (peak rarefactional pressure) to the dorsal posterior (DP) nerve to modulate the activity of motoneuron DE-3. Estimating nerve tissue to have a density of 1.03 g/cm^3^ (Mendez et al., 1960), and assuming the speed of sound in saline is approximately 1,507 m/s at 22°C (Goss and O’Brien, 1979), this pressure yields a spatial peak pulse average intensity (I_SPPA_) of 140 W/cm^2^. This intensity approaches, but does not exceed, the FDA limit for diagnostic use (190 W/cm^2^) (FDA, 2019). The maximum pulse amplitude applied to neuronal somata in our intracellular study (Collins and Mesce, 2020) was 225 kPa (I_SPPA_ = ∼ 16 W/cm^2^), though most recordings were lost due to electrode resonance at pressures ≤100 kPa (I_SPPA_ = ∼ 3 W/cm^2^).

The pulse energies applied in our studies: I_SPPA_ = 140 W/cm^2^; max I_SPPA_ = 16 W/cm^2^ are well in excess of those used in most FUS neuromodulation studies, particularly transcranial studies [e.g., Yoo et al., 2018 (4.2 W/cm^2^); Kim et al., 2019 (up to 61.5 mW/cm^2^); Tufail et al., 2010 (211.7 mW/cm^2^); Lee et al., 2016 (up to 14.3 W/cm^3^); Legon et al., 2014 (29.3 W/cm^3^)]. In Collins et al. (2021), comparable intensities were insufficient to modulate the activity of the spontaneously-firing DE-3 neuron, nor evoke spikes from any of the other neurons whose axons pass through the DP nerve. This is consistent with others’ findings that peripheral nerves, in comparison to central neural tissues, require much more energy to achieve an effect (Guo et al., 2022). Authors Wright et al. (2017) averaged intensities used to modulate peripheral nerves across studies, and calculated a mean intensity of 59 W/cm^2^, *versus* 3 W/cm^2^ in CNS studies. Notably, though our intensity was more than twice the average used to modulate peripheral nerve activity, we failed to observe results until pulse durations were lengthened to generate significant tissue heating.

In Collins and Mesce (2020), we applied short pulses of FUS at increasing pressures and intensities until we observed what we believed to be electrode resonance-induced depolarization of the resting membrane potential of identified neurons (Figure 1). At our lowest pressure tested (∼14 MPa, I_SPPA_ = 63 mW/cm^2^), parameters with which others have reported successful stimulation (Kim et al., 2019), none of the 12 impaled neurons in our study responded, either *via* changes in the voltage of the resting membrane potential, or by increasing neuronal firing. The full complement of parameters used in this study ran the gamut of those reported in the majority of transcranial studies (Pasquinelli et al., 2019), using intensities ranging from 63 mW/cm^2^ to 16 W/cm^2^, pulse durations from 100 to 300 ms, and both pulsed (30% duty cycle) and continuous FUS. Despite extensive attempts, the only modulation we were able to generate was artifactual, and resulted from FUS-induced electrode resonance, which could be compellingly replicated by micro-displacements of the recording electrode (Collins et al., 2021).

Despite our use of intensities in excess of what many other groups have found sufficient to modulate neuronal activity, it may be the case, paradoxically, that we did not go high *enough* to generate true mechanical effects. Work by other groups has revealed that mechanical neuromodulation may require the use of extremely high pressures, up to an order of magnitude greater than ours. In a preparation comparable to ours (Collins and Mesce, 2020), and using a comparable FUS frequency (1.1 MHz vs. our 960 kHz), Wright et al. (2017) were unable to modulate nerve activity using short pulses (negligible heating), even at extremely high intensities (4.2 kPa, 475 W/cm^2^). At a lower frequency (670 kHz), the authors were able to evoke compound action potentials starting at a threshold of 169 W/cm^2^. This lower frequency and high intensity correlated with inertial cavitation activity, which was reported to be the primary actuator of neural activation, but which caused significant damage in a minority of nerves tested. Recent publications by another group exploring the effects of FUS in mammalian systems also reported the need for extremely high energy to modulate peripheral nerve activity. Pressures of 11.8 MPa and 30 MPa, respectively, were used to elicit motor responses during stimulation of sciatic nerve in mice (Kim et al., 2020; Lee et al., 2020). With respect to the myriad publications describing activation of the cortex in mammals with much lower FUS intensities, perhaps modulation is not readily possible in the leech system at comparable intensities, even when targeting a single neuron *via* its soma (Collins and Mesce, 2020). However, it is much more plausible that prior reports of neuronal activation in mammalian systems have been influenced by incidental activation of sensory structures or other confounds, as will be discussed in the following section.

## The confounds of prior studies

Beyond the confounds posed by incidental activation of sensory systems, we recently showed that some recording modalities may influence FUS neuromodulation outcomes (Collins and Mesce, 2020). We found that experiments incorporating single-cell intracellular sharp recordings are vulnerable to depolarizing FUS-induced electrode resonance. Similar issues have been reported in other single-cell paradigms, including two-electrode voltage clamp in *Xenopus* oocytes (Kubanek et al., 2016), and patch clamp in mammalian cortical neurons (Tyler et al., 2018). Although not discussed in their study of FUS-activated TRAAK channels, the use of rigid recording pipettes located to the FUS field may have confounded the ionic currents they measured (Sorum et al., 2021). Importantly, depolarization induced *via* FUS in leech neurons, as previously reported (Dedola et al., 2020) and reproduced by us, could be fully replicated by micron-level movements of the recording electrode (Figure 1). The effects of FUS on spike frequency and amplitude were similarly reproducible. Our findings thus challenge the conclusions of studies that have utilized single cell electrophysiology to assess underlying FUS mechanisms, which have typically reported excitation/depolarization of the resting membrane potential, and have universally attributed such results to mechanical effects (Tyler et al., 2008; Prieto et al., 2018; Dedola et al., 2020).

Ultimately, we failed to observe evidence of mechanically-mediated FUS neuromodulation, despite targeting both somata and peripheral nerves, and modulating parameters including pressure, pulse duration, duty cycle, and (to a limited extent) frequency. Parameters that failed to yield significant (>1°C) tissue heating (e.g., short pulses) failed to modulate peripheral nerve activity (Collins et al., 2021), and effects elicited in neuronal somata appeared artifactual (Collins and Mesce, 2020). Possibly, the mechanical actions of FUS exerted more subtle effects that could have become perceptible over the course of hours or days, beyond the feasible recording window of our own study. Regardless, we remain convinced that the actions of FUS on non-mechanosensory neurons, at moderately low intensities (within FDA-allowable range for diagnostic use), are predominantly thermal. We are of the opinion that thermal neuromodulatory actions can be achieved more reliably and efficiently than mechanical ones; possibly, embracing and exploiting a thermal mechanism will thus likely expediate FUS technology’s transition from the lab to the clinic.

Additional studies are clearly warranted to establish that cortical and other CNS neurons are, indeed, preferentially susceptible to mechanical modulation due to their expression of mechanosensitive ion channels, including subtypes of Piezo and TRP channels, which appear to be mechanically activated by FUS (Prieto et al., 2018; Yoo et al., 2022). At least *in vitro*, compelling evidence exists that purely mechanical effects of FUS are possible (Prieto et al., 2013; Yoo et al., 2022). That said, although Piezo and TRP channels are expressed in cultured cortical neurons and possibly other CNS tissues, it remains unclear whether channel expression is sufficient to enable modulation of desirable neural targets *in vivo*, or whether increasing ion channel conductance is clinically desirable, as discussed below.

Piezo2 is highly expressed in dorsal root ganglion neurons, though Piezo1 and 2 mRNAs in brain are significantly lower than in other body tissues (Coste et al., 2010). Potentially further limiting these channels’ utility as a target, Piezo channels may play a significant role in pain pathology. Piezo2 is colocalized to nociceptors, and upregulation is hypothesized to contribute to hyperalgesia and allodynia (Volkers et al., 2014). Both Piezo1 and 2 are expressed in trigeminal sensory neurons, and excessive and repetitive activation of these channels is believed to contribute to migraine (Pietra et al., 2020).

TRP channels believed to be stimulated by FUS may face similar limitations, e.g. TRPC1, one of the TRP channels recently implicated in mechanically-mediated neuronal response to FUS (Yoo et al., 2022). While this channel does have widespread expression in the brain (Riccio et al., 2002), regions in which it is most highly expressed may not be especially clinically useful as a target for FUS stimulation; e.g., the cerebellum. Furthermore, increasing activation of TRPC1 channels may pose clinical risks. FUS has been shown to excite primary cortical neurons through a cascade initiated by an FUS-induced TRPC1-mediated calcium conductance (Yoo et al., 2022). Though not observed in association with ultrasound, increased neuronal calcium conductance can induce necrosis or apoptosis as a result of glutamate-induced excitotoxicity (Lau and Tymianski, 2010). Notably, increased calcium conductance *via* TRPC1 has been implicated as a primary actuator of glutamate-induced cell death (Narayanan et al., 2008).

Limitations in the expression patterns of canonical mechanosensitive ion channels in nervous tissues, and potential risks inherent in significantly increasing the activity of these ion channels, underscore the constraints associated with pursuing purely mechanically-mediated FUS neuromodulation. The following section highlights the likely mechanisms underlying thermal FUS neuromodulation, and emphasizes its potential clinical attributes while weighing its possible risks.

## Heat as a valuable and versatile actuator of ultrasound neuromodulation

Tissue absorbs FUS energy as heat, which is a well-established neuromodulator in its own right (Janssen, 1992). FUS-induced tissue heating can be potentiated by stimulation frequency, increasing duty cycle in pulsed applications, increasing stimulus durations, and increasing intensity. FUS at parameters that maximize thermal effects, e.g. high-intensity focused ultrasound (HIFU), is destructive, and has been used clinically to lesion noninvasively tumors of the prostate, uterus, and brain (Evans et al., 2007).

Some of the earliest investigations of FUS’s effects on neural firing utilized HIFU, and produced modulation replicable by increasing preparation temperature. FUS was found to block conduction of impulses in the peripheral nerves of earthworms, cats, monkeys, and humans at parameters that induced temperature increases of ≥17°C (mammalian preparations) or ≥6°C (earthworm), which could be replicated by focused heating of the nerves (Lele, 1963). In a cortical study, FUS-induced temperature increases of ≥7.5°C in rats caused an indirect modulation in the form of spreading depression, a phenomenon characterized by a period of neuronal depolarization followed by hyperpolarization (Ueda et al., 1977). In both studies, however, this thermal modulation was not without consequence. In the first study, nerve conduction block was accompanied by hemorrhage, ulceration, and significant skin damage when examined during transcutaneous applications (Lele, 1963); in the second study, cortical heating caused severe necrosis of neurons and glia at the FUS focus (Ueda et al., 1977).

While HIFU continues to present therapeutic opportunities in some neurological capacities (e.g., lesioning the ventral intermediate nucleus of the thalamus to treat essential tremor (Lipsman et al., 2013), or temporarily disrupting the blood-brain barrier to permit localized drug delivery (Mesiwala et al., 2002)), nondestructive low intensity applications (LIFU) are of primary interest to FUS neuromodulation researchers, as these applications present an opportunity to mimic noninvasively the effects of clinically successful electrical stimulation devices (deep brain stimulation devices, *etc.*) without the risks of surgery. LIFU applications, which typically use parameters that fall within the FDA-approved limits for diagnostic applications (FDA, 2019), can be used to generate moderate levels of tissue heating, which can nonetheless significantly impact neural firing.

### Factors that influence response direction and magnitude

In our study of the effects of 960 kHz FUS on the firing rate of a spontaneously-firing identified motoneuron in the medicinal leech, we found that short pulses (100–300 ms in duration) were insufficient to modulate neural activity, but much longer exposures, on the order of ten or more seconds, could induce reliable neuromodulation (Collins et al., 2021; Figure 2). These long durations facilitated the generation of significant tissue heating. In an attempt to determine whether the FUS-induced neuromodulatory effects on an identified motoneuron were thermal, we replicated the FUS-associated heating, which we estimated raised the temperature of our nerve by approximately 3.5°C, with two heating modalities: a 50 mW laser and a nickel-chromium wire device that heated when connected to a direct current source. The laser heated tissue by approximately 2°C, and the wire device heated by approximately 4.5°C.

Like FUS, both heating modalities induced compelling neuronal excitation and suppression, suggesting that net tissue temperature increase (2°C vs. 4.5°C) was not the sole determinant of any given neuromodulation outcome. For example, differences with respect to the percentage of trials that yielded each effect has suggested that intrinsic differences in the spatial or temporal thermal gradients of the two conditions can induce different bioeffects on our targeted nerve (Collins et al., 2021). In fact, these spatial and temporal gradients may be of greater significance than total temperature, at least within this 2.5°C range. We observed relatively more inhibition (*via* conduction block) with the 2°C laser than the 4.5°C wire device, a finding contrary to a more commonly reported trend of higher heat stimuli inducing inhibition, and lower heat stimuli inducing excitation. This finding suggests that spatial and temporal gradients are, in fact, the primary determinants of thermal neuromodulation outcomes within this range of temperatures (Figure 3).

### The neuromodulatory effects of moderate heating: The infrared neural stimulation connection

The mild-to-moderate increases in temperature observed in our leech nerve study can significantly impact neurophysiology. Much of what is currently understood about the neuromodulatory effects of non-noxious heat on nervous tissue derives from the infrared/optical literature. Like FUS, infrared neural stimulation (INS) generates spatially restricted heating of nervous tissue, and can induce both excitation and inhibition of neural activity (Cayce et al., 2014). INS enacts modulation through an as-yet poorly understood mechanism, but one that is believed to be photothermal, as opposed to photochemical or photomechanical (Zhao et al., 2016). Due to the relative wealth of INS studies compared to thermally-focused FUS neuromodulation studies, this literature will be called upon frequently to contextualize our hypothesized neuromodulatory mechanisms in the following sections.

### Thermal effects on neural activity at moderate temperatures: The spatial component

The leech, like all invertebrates, is an exothermic animal whose body temperature fluctuates with changes in environmental temperature. The animal’s nervous system maintains functionality across rapid shifts in temperature that can range in the tens of degrees; for example, when transitioning from land to water. Exothermic animals have evolved adaptations to maintain neural circuit activity across a wide range of body temperatures, which have been described extensively in the crab, *Cancer borealis*, whose stomatogastric ganglion (STG) is among the best characterized neural networks in any animal system. The STG produces a pyloric rhythm mediated by the orderly activation of select neurons (Marder and Bucher, 2007). Although pyloric rhythm frequency increases with increasing temperature, the phase relationships governing this rhythm are maintained by compensatory changes in input conductance, synaptic currents, transient outward currents, and hyperpolarization-activated inward currents (Tang et al., 2010). This feeding-related circuit thus maintains relatively normal functionality across temperature changes of at least 15°C (Tang et al., 2010; Soofi et al., 2014). Studies of temperature compensation in other invertebrates and exothermic vertebrates have reported similar findings, in which neural circuit frequencies increase upon exposure to increases in environmental temperature, but circuit functionality persists due to complementary temperature coefficients (Q_10_s) of opposing processes (Robertson and Money, 2012).

Given the leech’s ability to maintain neural firing across significant environmental temperature changes, owing presumably to similar compensation mechanisms to those described in other organisms (Robertson and Money, 2012; Städele et al., 2015; Städele and Stein, 2022), we were initially surprised that artificial temperature increases as low as 2°C could cause the dramatic modulation of neural activity we observed (Collins et al., 2021). One key difference between our heating modalities and those employed in most studies examining temperature compensation is the spatial gradient of the temperature increase. Most studies have examined the effects of global heating on neural function, typically *via* bath heating (e.g., Tang et al., 2010; Soofi et al., 2014), whereas our heating modalities generated spatially restricted heating ranging from a 1.2 mm diameter (laser) to an 8.5 mm diameter (wire device), with FUS heating a 6.8 mm diameter area of tissue (Collins et al., 2021). In a series of control studies, we also heated the bath temperature, enabling us to compare the effects of localized heating to global heating. We observed a strong trend whereby broader heating patterns were associated with a greater propensity towards an excitatory response, while more spatially restricted heating was biased towards an inhibitory response (Figure 3). The greater bias towards neural excitation with broader heating, including our exclusively excitatory results during bath heating [which mirrored results of prior bath heating experiments in leech (Romanenko et al., 2014), were reminiscent of reported increases in neural activity in response to global temperature increases reported in a wide range of exothermic animals (Robertson and Money, 2012).

### Thermal effects on neural activity at moderate temperatures: The temporal component

Our nerve study compared the effects of FUS to comparable heating induced by a laser and a wire device (Collins et al., 2021). While the magnitude of tissue heating generated by FUS fell between the two other heating modes, the rate of temperature increase differed markedly. The laser and wire device heated rapidly, with the first degree of temperature increase achieved at a rate of 0.35 C/s and 0.46 C/s respectively, as compared to a rate of 0.17 C/s with FUS. These differing temporal gradients are reflected in our neuromodulation data, with the peak modulation period (defined as the period of maximal difference in firing rate over baseline) occurring earlier in the laser and wire device trials in comparison to the FUS trials (peak effects began 10 s after the onset of the 30 s stimulus application for the laser and wire device, and 20 s after the onset of the stimulus in the FUS trials). The faster rate of heating of the two heat-only stimuli may also have enhanced the magnitude of the modulation achieved, defined as the mean firing rate during the peak modulation period normalized to the baseline firing rate. We observed more dramatic reduction in motoneuron firing with our rapidly heating modes, with a mean reduction of firing of 92% and 86% in our inhibitory laser and wire device trials, respectively, as compared to a mean reduction in firing of 43% in our inhibitory FUS trials (Figure 3).

### Potential molecular mechanisms of thermal neuromodulation

Temperature increases below the range at which significant tissue damage (e.g., protein denaturation) occurs have been proposed to modulate neuronal activity through several mechanisms, including increased membrane capacitance, and changes in ion conductance, which are argued to affect neuronal excitability *via* depolarization or hyperpolarization of the resting membrane potential. These processes are not mutually exclusive, and the net neuromodulatory outcome of tissue heating *via* ultrasound or other thermal stimuli may be dependent on the interaction of these mechanisms, whose relative contributions may be weighted by factors including the magnitude of temperature increase, spatial and temporal heating gradients, and the intrinsic characteristics of the target tissue (e.g., the types of ion channels expressed and thermal diffusion rates).

### Modulation of membrane capacitance

Changes in membrane capacitance have been proposed as an effector of FUS’s thermal effects on neuronal activity (Kamimura et al., 2020), and may be mediated by heat-induced membrane dimensional changes and displacement currents (Plaksin et al., 2018). Experimentally, short pulses of INS have been shown to increase the membrane capacitance of mammalian HEK cells and artificial bilayers, and to depolarize *Xenopus* oocytes though a presumably related mechanism, which operates independently of the presence of voltage-gated ion channels (Shapiro et al., 2012).

While there exists compelling evidence to support a capacitance modulation-based mechanism in INS (Zhao et al., 2016), the relevance of this mechanism to FUS applications remains unclear. INS studies have typically used short pulse durations that generate rapid and significant tissue heating (e.g., 10 ms pulses of up to 22°C of heating; Shapiro et al., 2012). In fact, it has been reported that the reliability of INS-evoked potentials begins to wane with pulse durations longer than 10 ms due to heat diffusion (Wells et al., 2007).

Achieving significant temperature increases on the order of milliseconds requires a very steep temperature gradient. One report of the modeled actions of INS on nerves revealed an inverse relationship between the rate of temperature increase and the magnitude of heating necessary to evoke action potentials (Fribance et al., 2016). In this model, rapid increases in temperature sufficient to depolarize neurons and elicit action potentials *via* increases in membrane capacitance required a 6.6–11.2°C increase in <3 ms, though lower temperatures were sufficient with faster heating kinetics. Conservatively, this equates to a 2,200 C/s heating rate required to elicit firing through a membrane capacitance-mediated mechanism. This *vastly* exceeds the heating rate generated by FUS in our nerve study, as well as that of our heat-only modalities (Collins et al., 2021). Given this orders-of-magnitude difference, it is unlikely that heat-induced increases in membrane capacitance were a primary actuator of the minority of FUS trials in which we observed excitation, and may explain why other groups who have used FUS to thermally modulate neural activity have failed to evoke activity in a manner comparable to what has been reported in the INS literature (e.g., Darrow et al., 2019). It is possible that future thermal FUS neuromodulation applications could utilize parameters sufficient to elicit the rapid, significant heating necessary to evoke activity by increasing membrane capacitance, although the high intensities required may pose substantial safety risks.

### Thermal gating of ion channels

Heat can also modulate neuronal activity by altering ion conductances. The temperature sensitivity of biological processes can be quantified by Q_10_ values, which describe the ratio of the rate of the process at two temperatures separated by 10°C. With respect to ion channels, this can refer to the rate of channel gating or channel conductance. Although all ion channels have some degree of thermosensitivity, ion channels are not typically categorized as thermosensitive unless they have a Q_10_ ≥ 2 (Hille, 2001).

One class of ion channels frequently implicated in a neuronal response to heat is TRP, a family of cation-nonspecific ion channels with well-characterized roles in mediating responses to sensory stimuli including changes in temperature (Clapham, 2003). Highly thermosensitive TRP channels, such as members of the vanilloid (TRPV) subfamily, can have Q_10_s ≥ 20 (Clapham, 2003). TRPV channels are highly expressed in thermosensitive sensory neurons of the dorsal root ganglia (DRG), and are also reportedly found in most CNS tissues including the cortex, where they may regulate neuronal responses to changes in osmolarity and pH in addition to temperature (Kauer and Gibson, 2009). These channels have been explored as effectors of INS. Infrared light increases single-channel activity of TRPV channels expressed in *Xenopus* oocytes (Yao et al., 2009). TRPV4 channels have been shown to mediate INS-evoked potentials in vestibular and retinal ganglia neurons (Albert et al., 2012), and TRPV1 channels in sensory fibers of the vagus nerve are necessary for infrared photostimulation (Rhee et al., 2008). The contribution of TRPV channels to thermal neuromodulation in non-sensory tissues is less clear, though one study of the effects of INS on cultured hippocampal primary neurons demonstrated an indifference of effects to Ruthenium Red, a non-specific TRPV channel blocker (Feyen et al., 2016). Given their well-established responsiveness to thermal stimuli, TRPV channels are likely activated upon stimulation with heat-generating FUS or other heating modalities, introducing depolarizing Na^+^ and Ca^2+^ currents in neurons. The net thermal neuromodulation outcome, however, will be highly dependent on the extent to which TRP channels are expressed in target tissues, and whether their actions are enhanced or counteracted by the activation of other thermosensitive ion channels.

Other classes of ion channels implicated in thermal neuromodulation are the voltage-sensitive ion channels, including Na_V_ and K_V_. Experimentally, increased temperature is associated with an increase in the rate constants of ion conductances mediated by these channels in umyelinated axons and at the Nodes of Ranvier of myelinated nerves (Frankenhaeuser and Moore, 1963). Increases in the gating kinetics of these channels are consistent with established heat-induced neurophysiological changes beyond net increases or decreases in neuronal firing. These include reductions in spike width and amplitude, and increases in conduction velocity (Hodgkin and Katz, 1949; Haveman et al., 2004), which have been reported in the INS (Xia and Nyberg, 2019) and thermal FUS literature (Tsui et al., 2005). Whether these channels are the primary effectors of thermally-mediated changes in firing rates at the moderately low temperatures (<5°C) used in our study (Collins et al., 2021) and others (e.g., Darrow et al., 2019), however, is less clear.

Mechanically-increased Na_V_ conductance has been proposed to underlie FUS-induced excitation (Tyler et al., 2008; Tufail et al., 2010), and FUS has been shown to directly increase heterologously-expressed Na_V_ conductance (Kubanek et al., 2016). A more recent study showed that Na_V_ was not readily mechanically activated at parameters sufficient to activate mechanosensitive Piezo1 channels (43 MHz, up to 90 W/cm^2^ I_SPPA_), but Na_V_ could be activated thermally (Prieto et al., 2018). Though not exceptionally thermosensitive (Q_10_ ≤ 1.5), increases in temperature steadily increase Na_V_ conductance (Milburn et al., 1995), which could sufficiently depolarize neurons to elicit firing. Na_V_ may thus contribute to increased firing in the minority of our thermal FUS trials that resulted in excitation (Collins et al., 2021).

Na_V_-mediated effects could also underlie thermal inhibition. INS researchers have proposed that infrared’s inhibitory effects, which include axonal conduction block, which we observed in our study during FUS-mimicking laser-induced heating (Collins et al., 2021), may be driven by prolonged inactivation of Na_V_ (Peterson and Tyler, 2014). Recent work has shown, however, that INS-induced conduction block persists in the presence of the Na_V_ blocker tetrodotoxin (TTX) (Ganguly et al., 2019a), suggesting Na_V_ channels are not the primary mediators of heat-induced reductions in firing rates. These channels may nonetheless affect other neurophysiological changes, including the reduction of spike amplitudes, which have been shown in primary cortical neurons to be mediated primarily by the increased inactivation of Na_V_ (Yu et al., 2012). This is consistent with our own data, in which we sometimes observed stimulus-induced truncation of spike waveforms regardless of the effects on firing rate as recorded proximally to the region of conduction blockade, suggesting that this truncation is enacted independently of the mechanisms driving action potential propogation (Morgan Collins, personal communication).

Rather than a Na_V_-mediated mechanism, there exists compelling evidence that heat-induced conduction block is primarily caused by an increased potassium conductance. Hyperthermia elicits a rapid increase in extracellular potassium in invertebrate and mammalian systems, and thermal conduction block may be mimicked by artificially increasing extracellular potassium (Wu and Fisher, 2000; Money et al., 2009). Recent studies suggest a primary source of this increased potassium conductance may be K_V_. Although TTX was not found to affect heat-induced conduction block in nerves in *Aplysia*, the phenomenon was shown to be greatly reduced by tetraethylammonium (TEA), a K_V_ antagonist (Ganguly et al., 2019a). Additional modeling work has demonstrated that thermal conduction block is likely a K_V_-mediated phenomenon in which rapid channel activation and increased channel conductance hyperpolarizes neurons (Ganguly et al., 2019b).

Although heat-induced increased K_V_ conductance is likely a primary contributor to conduction block, the contribution of these channels to other forms of thermal inhibition is less clear. We collected limited data with a high-heat (∼10°C) transducer compatible with intracellular recording that revealed a hyperpolarization of the resting membrane potential (Morgan Collins, personal communication). While these data should be interpreted with caution given the potential for FUS-induced electrode resonance artifact in intracellular paradigms (Collins and Mesce, 2020), there exists in the literature support for this alternative mechanism, particularly in cases of more profound temperature increase (e.g., ≥10°C). Consistent with our results, which were generated with broader, slower, and higher-magnitude FUS-associated heating than our conduction block-inducing laser, bath heating in *Aplysia* hyperpolarizes neurons by a typical rate of 1–2 mV/°C (Carpenter, 1967). This hyperpolarization is consistent with the Nernst potential changing and a potential increased K_V_ activation. It may also be driven or potentiated by a separate, but complementary, potassium conductance through two-pore K^+^ channels (K_2P_).

K2P are voltage independent leak channels that assist in the maintenance of the resting membrane potential (Enyedi and Czirják, 2010). Ultrasound increases conductance of the K2P, TREK-1, TREK-2, and TRAAK (Kubanek et al., 2016), potentially *via* a thermal mechanism (Prieto et al., 2020). Many subtypes of K2P are thermosensitive, including TREK and TRAAK, which are expressed widely in both the central and peripheral nervous systems (Schneider et al., 2014). Thus, thermal FUS-induced increased conductance of these channels would hyperpolarize neurons, bringing the membrane potential closer to the K^+^ equilibrium potential to inhibit neuronal firing. Such temperature-related hyperpolarizations have been observed in the neurons of the STG (Städele et al., 2015).

Voltage-gated and ion-activated calcium channels may also contribute to thermally-mediated FUS neuromodulation. In our studies, however, both neuronal excitation and conduction block persisted when the preparation was bathed in Ca^2+^-free saline (Collins et al., 2021). This does not preclude the potential for Ca^2+^ influx-mediated modulation or potentiation of effects, but it does suggest that calcium channels were neither the primary initiators nor effectors of thermal neuromodulation in our heating paradigms.

Finally, heat may also increase conductance of ligand-gated ion channels by facilitating synaptic vesicle release from presynaptic terminals (Wang et al., 2014). Increases in temperature lower energetic barriers to SNARE protein-mediated fusion of synaptic vesicles to neuronal cell membranes (Gao et al., 2012). Thermal effects on synaptic transmission may be further potentiated by cationic TRPV channels, which are reportedly present on presynaptic terminals in several brain regions (Kauer and Gibson, 2009). The neuromodulatory outcome of thermal FUS-induced increased synaptic activity would be dependent on whether released neurotransmitters were excitatory or inhibitory.

## Conclusion

As the preceding sections attest, the thermal actions of FUS on neurons are likely multimodal. The direction and magnitude of the resultant neuromodulation is likely reliant on a complex interplay of changes in membrane capacitance, ion channel conductance, and membrane potential. These effects, which may be complementary or opposing, are likely further influenced by additional modulating factors beyond the scope of this review. These include long-term effects, such as changes in neuronal gene expression, which may be initiated by Ca^2+^ influx (West et al., 2001), a known bioeffect of FUS stimulation (Tyler et al., 2008; Zhou et al., 2017; Yoo et al., 2022).

The leech model, in particular, has been an exemplary preparation in which to gain mechanistic insights into the bioeffects of FUS. The leech’s large, physiological robust and identifiable neurons have enabled electrophysiological investigations into FUS’s neuromodulatory mechanisms at the single-cell level. The spontaneous firing properties of neurons including DE-3 and the Retzius neuron have permitted analyses of FUS’s effects on natural–*versus* evoked–firing activity. The size and flexibility of the preparation enable investigative paradigms that are challenging in other systems, including simultaneous intracellular somatic and extracellular axonal recording from the same cell. The extensive literature describing leech neuronal ionic conductances will also facilitate predictions of the technology’s likely underlying mechanisms. Finally, we intentionally chose to assess the effects of FUS on a motoneuron, a class of neurons not “built” to respond to mechanical stimuli. FUS-mediated effects on motoneurons, which are unlikely to express canonical mechanotransductive ion channels, may be more representative of other non-sensory cell types, including interneurons.

With respect to clinical applications, it is unlikely that a single, “one size fits all” FUS therapy will comparably modulate neuronal activity across tissue types, given intrinsic differences in ion channel expression and distribution present in, for example, axons *versus* neuronal somata. Responses will also vary dependent on the types of connective tissue surrounding neuronal targets, which may influence the rate and magnitude of heat generated due to associated factors including thermal diffusion rates (Wells et al., 2007). Similarly, the actions of glia will likely prove significant in determining the type and duration of heat-induced neuromodulation, particularly in inhibitory contexts, wherein effects correlate with an increase in extracellular potassium. The extent to which (K^+^)_o_ is elevated as a consequence of heating will be dependent, in part, on rates of astrocytic uptake (Walz, 2000). This is of particular relevance for cortical FUS applications, as sharp increases in (K^+^)_o_ can elicit spreading depression, a phenomenon associated with migraine and other neurological disorders (Ayata and Lauritzen, 2015), which have been reported to occur in response to high-heat cortical FUS applications (Ueda et al., 1977; Koroleva et al., 1986).

Despite heat’s complicated array of actions on the nervous system, patterns in neural responses to heat are apparent and may be used to inform the design of thermal FUS neuromodulation therapies biased to generate a desired response, be it neuronal excitation or inhibition. Within the range of temperatures used in our studies (2–4.5°C, which are tolerable over durations <1 h in mammalian systems (Haveman et al., 2005), FUS parameters that yield broader heating may promote an excitatory response. Such responses may be driven by the activation of thermosensitive sensory structures in surrounding tissue or by broad increases in the kinetics of circuit-mediating ion channels with complementary Q_10_s, which may be potentiated by an increased Na_V_ conductance. Importantly, finer FUS foci may inhibit neurons through the localized induction of hyperpolarizing potassium currents through K_V_ and K_2P_ channels. In contrast, adjusting FUS parameters to promote sharper temporal gradients in heating, which mimic those of INS, may more readily enable excitation with finer foci. Finally, there remains great potential to use FUS as a *bimodal* neuromodulatory technology. Clearly, future research should explore the ways in which mechanical effects can potentiate thermal ones, and *vice versa*, e.g., in tissues expressing both mechanosensitive and thermosensitive ion channels.
